# Educating Young Adults about Opioid Misuse: Evidence from a Mass Media Intervention

**DOI:** 10.3390/ijerph19010022

**Published:** 2021-12-21

**Authors:** Jessica M. Rath, Siobhan N. Perks, Donna M. Vallone, Alexis A. Barton, Daniel K. Stephens, Bethany Simard, Elizabeth C. Hair

**Affiliations:** 1 Schroeder Institute at Truth Initiative, Washington, DC 20001, USA; snmperks@gmail.com (S.N.P.); dvallone@truthinitiative.org (D.M.V.); alexis.a.barton@gmail.com (A.A.B.); dstephens@truthinitiative.org (D.K.S.); bethany.simard@gmail.com (B.S.); ehair@truthinitiative.org (E.C.H.); 2 Department of Health, Behavior and Society, Johns Hopkins Bloomberg School of Public Health, Baltimore, MD 21205, USA; 3 Department of Behavioral and Community Health, University of Maryland School of Public Health, College Park, MD 20742, USA; 4 School of Global Public Health, New York University, New York, NY 10003, USA

**Keywords:** prescription opioid misuse, public health media campaigns, young adults

## Abstract

The US opioid epidemic is a serious public health problem. Rates of opioid misuse and dependence are highest for young adults ages 18–25. Prevention strategies that reduce prescription opioid misuse while decreasing stigma around dependence and treatment are critical components of addressing the epidemic. The Truth About Opioids, a mass media public education campaign, was designed to prevent opioid misuse and dependence among young adults. This study examined the intervention’s effectiveness to shift opioid-related knowledge, attitudes, and beliefs within targeted designated market areas (DMAs) over time. A sample of young adults (*N* = 1434) in DMAs with varying levels of media exposure was surveyed at baseline (June–September 2019) and post-intervention (July–August 2020). Logistic regression assessed associations between campaign awareness and campaign-targeted knowledge and attitudes, controlling for baseline variables. Those with any awareness had significantly higher odds of campaign-targeted opioid-related knowledge (versus no awareness) (low awareness OR = 1.52 (95% CI: 1.04, 2.24); high awareness OR = 2.47 (95% CI: 1.58, 3.87)). Those with campaign awareness were also more likely to report lower levels of opioid-related stigma and higher intentions to share information and talk to a friend about the epidemic. Mass media public education campaigns can help influence young adults’ opioid-related knowledge and attitudes.

## 1. Introduction

The opioid epidemic is a serious public health problem that continues to plague communities across the US. This epidemic stems from the misuse of prescription opioids (i.e., taking a prescription in a manner or dose other than prescribed, or without a doctor’s prescription) and has evolved to include the misuse of illicit (e.g., heroin) and synthetic (e.g., fentanyl) opioids in recent years [1]. Survey data consistently show that rates of opioid misuse and dependence are highest for young adults of ages between 18 and 25 years. According to the National Survey on Drug Use and Health, in 2018, about 5.6% of US young adults reported past-year opioid misuse [2], with significant variation in use and overdose rates by state [3]. High misuse rates are particularly concerning since those who misuse prescription opioids are more likely to transition to using heroin [4,5].

To help reduce trial and progression to established opioid use, prevention strategies including policies, public education campaigns, and school-based educational efforts have been identified as potentially effective approaches [6,7]. Experts suggest that prevention efforts should aim to increase knowledge of the risks associated with opioid misuse and decrease stigma against those suffering from opioid misuse [8,9]. Decreasing stigma about opioid dependence among the general population can help support those suffering with misuse or addiction to engage with treatment opportunities more comfortably. Decreasing stigma can also serve to increase broad public support for establishing policies and programs that can facilitate recovery.

One such prevention effort is The Truth About Opioids, a mass media campaign for young adults that delivered broad public education messages through television and digital media, including social media and video streaming platforms. Drawing from previous experience with successful anti-tobacco efforts for youth and young adults, Truth Initiative designed and implemented this public education effort to shift key knowledge, attitudes, and beliefs related to opioid misuse and dependence, which, in turn, prompt changes in intentions and behaviors [10]. According to the Centers for Disease Control and Prevention (CDC), when campaign messages are aired at sufficient levels of reach among the target audience, one can expect changes in campaign-targeted knowledge and attitudes in 6 to 12 months, and changes in behaviors in 12 to 24 months after campaign launch [11].

Campaign messages were developed using a rigorous formative research process, which included qualitative studies with addiction professionals and people struggling with opioid dependence, and iterative rounds of quantitative studies with young adults in the campaign’s target population. Specifically, the Hornik and Woolf method was used to identify initial message concepts, which were then tested in forced-exposure studies for perceived effectiveness, likability, and relevance [12,13]. Findings from the formative research phase confirmed the effectiveness of authentic, real-life stories of those impacted by opioid misuse. As a result, these messages featured true stories of young people whose dependence on opioids led them to take dramatic and harmful actions, as well as the story of a young woman as she began her journey in a treatment and recovery program. Using the personal stories of people who are like the young adults in the campaign’s target audience is important for capturing attention in a crowded media landscape. Early evidence of the messaging strategy was tested in Rhode Island, where data indicated messages significantly increased knowledge about misuse and increased empathy for those with opioid dependence [14]. Findings from the Rhode Island study prompted an additional pilot study with geographic variation among cities with high prescription opioid misuse, high overdose rates, and a longer intervention time period. This pilot study examined the short-term effects of The Truth About Opioids campaign to shift targeted knowledge, attitudes, and beliefs regarding opioid misuse and dependence. The research question was, “Does awareness of The Truth About Opioids campaign increase campaign-targeted knowledge and attitudes about prescription opioid misuse, and intentions to share information about the opioid epidemic?”

## 2. Methods

### 2.1. Intervention

The messaging strategy for The Truth About Opioids focused on telling the real-life stories of those struggling with opioid dependence. These specific narratives highlighted how opioid misuse and dependence can significantly alter the otherwise bright futures of young people. By highlighting the effects that opioid misuse can have on typical life milestones (e.g., graduation or senior night), messages were intended to evoke strong emotional responses among the young adult target population. Messages also chronicled a young woman’s journey through treatment for opioid dependence at an in-patient facility. Each message included a fact tagline: ‘Opioid dependence can happen in just five days,’ and a call-to-action prompt for fact exploration and conversations: ‘Know the Truth, Spread the Truth.’ Media messages were aired on television and digital media platforms preferred by young adults from September 2019 to June 2020. The goal was to reach over 90% of this population through a combination of local television and online platforms such as Spot TV, Hulu streaming, Search, Twitter, Snapchat, Facebook, and YouTube. YouTube performed the best, reaching 95% of the targeted 18 to 34-year-olds.

### 2.2. Sample

The sample for this longitudinal study was drawn using a commercially available list of cell phone numbers (obtained from Marketing Systems Group) with area codes associated with six designated market areas (DMAs) across three states. The six DMAs included Fort Wayne, Indiana; South Bend, Indiana; Greensboro, North Carolina; Charlotte, North Carolina; Knoxville, Tennessee; and Memphis, Tennessee, which reflected areas with high prescription opioid misuse and overdose rates, and limited population-based prevention actions. Sampled cell phone numbers were sent an SMS text message inviting them to participate in the study and a link to complete the questionnaire online. Any respondents who reported living outside of counties in the targeted DMAs or were outside of the target age range of 18–34 years were not surveyed. Of those that initially agreed to participate, 36% were eligible for the study. The surveys took approximately 25 min to complete, and participants were compensated $10 for each completed survey. ICF was the data collection vendor for this study and is a global advisory and digital services provider. Truth Initiative only received de-identified data from ICF. ICF is certified by the Council of American Survey Research Organization (CASRO) Institute for Research Quality for meeting ISO 20,252 quality standards. The study protocol was approved by the ICF internal Institutional Review Board (Project number: 190308.0.001.00).

A sample of 4765 young adults (ages 18–34 years) completed the baseline data collection in June through September 2019, before the campaign aired. The post-intervention survey data (*N* = 1434) were collected in July and August 2020, after the campaign finished airing. This pre-to-post attrition rate was higher than other studies; this may have occurred given that the post-intervention survey occurred during the COVID-19 pandemic. Moreover, the attrition may have been influenced by the study’s SMS text-based contact procedures. A total of 1,331,225 texts were sent. Text-based recruitment is a relatively new method which incurred some unanticipated problems. For example, the text to complete rate was high at 1738 texts sent per complete, which could have been due to the evolving sophistication of carrier blocking. It is important to note that while the number of texts sent is known, the number of texts received by potential respondents is unknown due to carrier blocking. Daily completion rate tracking indicated that on some days the text per completion rate was as low as 442 texts per complete, while on other days it was as high as 16,748 texts per complete. Overall, since SMS-based study efforts are a relatively novel method of engaging young people for research study participation, estimates for efficacy regarding recruitment and retention are still being established. However, even with a high attrition rate, an attrition analysis comparing those who responded to both baseline and post-intervention surveys, to those who participated in baseline only, demonstrated that participants lost to attrition were not systematically or significantly different in demographic characteristics or baseline attitudes than those who completed the post-intervention data collection.

We computed post-stratification adjustment factors separately for each DMA. These factors use the known population totals by aggregating county totals along demographic dimension of age groups, sex, and race/ethnicity obtained from the U.S. Census American Community Survey 5-year data for each county comprising each DMA. The population totals for these demographic variables were used in the post-stratification process. We assessed the variability of the final weights both within each DMA and across all DMAs. All design effects (DEFF) were less than 1.7. DEFFs of 2.0 (or less) are considered good in the sense that survey sampling variances are less than twice those that would be attained with a simple random sample of the same size.

### 2.3. Measures

#### 2.3.1. Campaign-Targeted Outcomes

This study was designed to assess individual-level changes in campaign-targeted knowledge, attitudes, and beliefs (KABs) over time, which were measured both at baseline and post-intervention. Most items were measured using a five-point agreement scale, with response options: strongly disagree, disagree, neither agree nor disagree, agree, and strongly agree. Responses were dichotomized so that those who responded ‘agree’ or ‘strongly agree’ were categorized as having agreement with the item. Campaign-targeted knowledge was measured as agreement with the statement ‘Opioid dependence can happen in just five days;’ stigma was assessed with the item ‘Someone like me could become dependent on prescription opioids.’ Risk attitudes of prescription opioid misuse were assessed with the item, ‘How much do you think people risk harming themselves if they use prescription opioids once or twice without a doctor telling them to?’ Response options included: no risk, low risk, moderate risk, high risk, and very high risk. Participants who responded, ‘moderate risk,’ ‘high risk,’ or ‘very high risk’ were categorized as reporting moderate/high risk perception.

Response to the call to action ‘Know the Truth, Spread the Truth’ was measured with two items which assessed the extent to which respondents would seek and share opioid-related information including, ‘How likely are you to do each of the following?: “Look up information about the opioid epidemic” and “Talk to a friend about the opioid epidemic.”’ Response options included very unlikely, somewhat unlikely, somewhat likely, and very likely. For analyses, participants who responded ‘somewhat likely’ or ‘very likely’ were categorized as likely to seek and share information.

#### 2.3.2. Outcomes Not Targeted by the Campaign

Items which do not reflect the campaign messaging strategy were also added to provide further evidence that changes in attitudes can be attributed to the campaign rather than other opioid-related efforts. Examining shifts in these opioid-related items serves as a validity check. Specifically, a non-targeted attitude was assessed with the item ‘Using medication to treat opioid dependence is like using medication to treat other chronic diseases like diabetes,’ for which responses were measured using a five-point agreement scale and were dichotomized so that those who responded ‘agree’ or ‘strongly agree’ were categorized as having agreement. The other non-campaign-targeted outcome was related to risk attitudes of illicit opioid misuse, which was measured with the item, ‘How much do you think people risk harming themselves if they try heroin or fentanyl once or twice?’ Response options included: no risk, low risk, moderate risk, and high risk, and participants who responded, ‘moderate risk,’ ‘high risk,’ or ‘very high risk’ were categorized as reporting moderate/high risk perception.

#### 2.3.3. Campaign Awareness

Awareness of the campaign was measured in the post-intervention survey. Participants were presented with collages of screenshots of the various ads and were asked, ‘These images come from multiple ads. How many times have you seen one of these ads, or one like it, recently?’ Response options were never (0), rarely (1), sometimes (2), and often (3). Responses were summed to produce values between 0 and 6. Scores of 0 or 1 were coded as no campaign exposure, scores of 2 or 3 were coded as low exposure, and scores of 4 or higher were coded as high exposure.

#### 2.3.4. Prescription Opioid Misuse

A respondent’s history of prescription opioid misuse in the past 30 days was measured with the following yes/no item: ‘Have you taken prescription opioids without a medical professional prescribing them to you in the past 30 days?’ Opioid misuse in a respondent’s social network was ascertained with four yes/no items about friends or family members using prescription opioids in the past 6 months. Specifically, participants were asked, ‘To the best of your knowledge, in the past 6 months, has a [family member who lives with you; family member who does not live with you; friend/roommate who lives with you; friend who does not live with you] used prescription opioids without a medical professional prescribing them?’ Respondents who answered ‘yes’ to at least one of the four items were categorized as having a friend or family member misuse prescription opioids in the past 6 months.

#### 2.3.5. COVID-19-Related Items

Additional survey items were added to the post-intervention survey to help account for issues related to the COVID-19 pandemic, including the personal challenges to daily life and its effect on mental health. Items included: ‘Which of the following changes to your daily life have you experienced because of the public health crisis resulting from coronavirus [COVID-19]?’ Respondents could select from a list of 12 possible issues including loss of income, virtual school or work, and food or housing insecurity (scale range = 0–12).

#### 2.3.6. Demographics

Demographic variables included participant age (18–24 years; 25–34 years), gender (male; female; transgender or other gender identity), race/ethnicity (non-Hispanic White; non-Hispanic Black; Hispanic/Latinx; non-Hispanic some other race), and subjective financial situation (can pay for basic expenses with more than a little left over; can pay for basic expenses with a little left over; can pay for basic expenses with nothing left over; can’t pay for basic expenses) [15].

#### 2.3.7. Psychosocial Characteristics

Depression symptoms were measured using two items indicating past 6-month frequency of sleep trouble and depressed mood [16]. Responses were averaged to form a single score that ranged from 0–3. Anxiety and stress were measured together using the mean response to items from the Anxiety and Stress subscales of the DASS-21 [17]. Sensation seeking was measured with the eight-item Brief Sensation Seeking Scale, and responses were averaged to form a single score ranging from 1–5 [18].

#### 2.3.8. County-Level Overdose Rates

To help control for contextual factors related to opioid misuse, drug overdose death rates for the targeted DMAs were obtained from the University of Wisconsin Population Health Institute’s County Health Rankings 2020 dataset. Rates were derived from the National Center for Health Statistics Mortality Files and defined as average number of prescription or illicit drug poisoning deaths per 100,000 from 2016–2018. Counties with fewer than 10 drug poisoning deaths were reported as missing in the dataset and were set to 0 for the analysis.

### 2.4. Analytical Approach

The analysis was conducted using a sample of individuals from all targeted DMAs (*N* = 1434). Weighted means, standard errors, and frequencies were calculated to examine sample characteristics. Weighted frequencies of awareness, overall and by age group were estimated to examine potential differences. Pearson’s Chi-Square Test of Independence, corrected for survey design, resulting in F statistics [19], was used to test whether the differences in awareness were statistically significant. Logistic regression models were estimated to examine the association between campaign awareness and changes in campaign targeted KABs post-intervention, controlling for baseline KABs and other covariates. Models used post-stratification weights to generalize to DMA-level populations of 18–34-year-olds. All analyses were performed using Stata 15.1.

## 3. Results

Sample characteristics for those completing both the baseline and post-intervention survey (*N* = 1434) are presented in Table 1. Most participants were between the ages of 25 and 34 (57.7%), female (53.8%), non-Hispanic White (70.3%), and reported being able to pay for basic expenses (95.4%). Most participants reported no misuse of opioids in the past 30-days (95.3%) and did not have a friend or family member misuse opioids in the past six months (78.2%).

Campaign awareness is described in Table 2. In the analytic sample, 26.0% of participants had high campaign awareness, 30.0% had low awareness, and 44.1% had no awareness. Awareness was significantly higher among the younger age group (18–24-year-olds) than the older group (25–34-year-olds) based on Pearson’s Chi-Square Test of Independence. Specifically, nearly two-thirds of 18–24-year-olds (63.7%) had some campaign awareness compared to about half (50.4%) of the 25 to 34-year-olds. There was low awareness (between 13–20% aware) of other campaigns running simultaneously including a television/digital advertisement in Tennessee and a Facebook-only prevention campaign targeting adults nationally.

Table 3 presents logistic regression models which examine the relationship between campaign awareness and each of the knowledge and attitudinal outcomes, controlling for DMA. Compared to those with no awareness of the campaign, participants with both low and high levels of campaign awareness had higher odds of agreeing with the knowledge statement, ‘Opioid dependence can happen in just five days’ (OR = 1.52, 95% CI: (1.04, 2.24); OR = 2.47, 95% CI: (1.58, 3.87), respectively). High campaign awareness was positively associated with endorsing the destigmatizing attitude, ‘Someone like me could become dependent on prescription opioids.’ Specifically, participants with high campaign awareness had 1.59 (95% CI: 1.06, 2.40) times greater odds of agreeing with the statement compared to those with no awareness.

Campaign awareness was also positively associated with both intentions to seek and share additional opioid-related information. Compared to no campaign awareness, both low and high campaign awareness were associated with greater odds of reporting high likelihood to look up information about the opioid epidemic (OR = 1.56 (95% CI: 1.11, 2.21) and OR = 1.75 (95% CI: 1.21, 2.54), respectively). Higher campaign awareness was associated with greater odds of reporting likelihood of talking to a friend about the opioid epidemic compared to no awareness (OR = 1.93 (95% CI: 1.33, 2.81)).

No statistically significant associations were observed between campaign awareness and risk attitudes of prescription opioid misuse. In addition, no statistically significant associations were observed between campaign awareness and the attitudes on which the campaign did not message (i.e., using medication to treat opioid dependence is like using medication to treat chronic diseases; and risk of illicit opioid misuse).

## 4. Discussion

These study findings provide evidence of the effectiveness of a mass media campaign to help influence opioid-related knowledge and attitudes at the population level. According to health behavior change theory, changes in attitudes precede changes in intentions and ultimately behavior change [10]. Awareness of The Truth About Opioids campaign was associated with greater knowledge of the fact that opioid dependence can happen in just five days, decreased stigma, and an increased likelihood to seek and share opioid-related information. This is noteworthy because baseline attitudes/behaviors were significant predictors in the models and follow public health theory of behavior change. Awareness of the campaign was not associated with the risk attitudes of prescription opioid misuse, which may reflect the relatively short nine-month campaign duration. Other attitudes that were not specifically targeted by the campaign messages were also not found to significantly change during the study period. These attitudinal changes are consistent with the CDC estimates of public education campaign effects, which indicate that changes in campaign-targeted knowledge and attitudes are expected to occur in 6 to 12 months after campaign launch [11].

Study results are consistent with preliminary research findings from other state and local opioid misuse prevention campaigns [20,21,22]. The CDC has also launched a public education campaign called Rx Awareness to raise awareness of the opioid epidemic and lower prescription opioid misuse among young adults. Preliminary evaluation analyses indicated that consumers exposed to the campaign reported higher awareness and knowledge of the risks and dangers of prescription opioids and had higher intentions to seek information or talk to others about prescription opioids [3].

Communicating about substance misuse is a complex and challenging task given the variation in substance use risk and pathways to other product use, particularly among young people. To help prevent early trial and experimentation, this phase of The Truth About Opioids intervention focused on misuse of prescription opioids given the evidence that those who misuse prescription opioids are more likely to eventually transition to using illicit opioids, along with other substances [4,5,23]. Those who misuse prescription opioids are also at greater risk of overdose and death [4,23]. Public education efforts focused on illicit substance use may also help raise awareness of the need for effective treatment, while reducing stigma for those who suffer from addiction disorders.

This campaign was being aired as COVID-19 emerged as an international pandemic with mandatory stay-at-home rules across the country. As a result, many schools and universities closed, and youth and young adults relied on virtual gatherings for social interaction rather than in-person events. These changes in daily life resulting from quarantine and social distancing have been found to increase stress and negative mental health outcomes, which, in turn, are likely to prompt increases in substance use among high-risk groups [24,25]. Although this analysis attempts to control for COVID-related issues, evidence of how, and to what extent, the COVID-19 pandemic may be exacerbating opioid misuse remains unclear [24,26].

Although this longitudinal study has many strengths, it is not without limitations. First, the study’s relatively short nine-month duration limits the likelihood of detecting behavior change, which typically occurs between 12 and 24-months post-campaign launch. It is also possible that not all effects in knowledge and attitudes were unfolded in the short time frame. Second, the higher-than-average attrition of the post-intervention sample may have introduced some sample bias. Given the short time frame and the high attrition rate, the effects presented may be conservative. Third, sample bias may have been introduced from drawing the sample from a commercially available list of cell phone numbers, rather than using an address-based sample. Fourth, it is possible that those who completed the baseline survey were more susceptible to the campaign, and thus overestimated their awareness in the post-intervention survey. Fifth, since this was a pilot study, there is a potential lack of generalizability to young adults outside the targeted DMAs. Finally, an unanticipated limitation of this intervention and its evaluation was in the alignment of the end of the media intervention and post-intervention data collection with the COVID-19 pandemic. Despite our efforts to account for some of these factors, we cannot eliminate the potential confounding effect of the COVID-19 epidemic on opioid-related outcomes.

Findings from this mass media campaign suggest that public education delivered at a national level could positively impact millions of young people. Using similar parameters of media delivery, this type of campaign could potentially reach 54 million young adults and help increase knowledge and attitudes related to opioid misuse and dependence among 6 million young people. Given the devastating consequences of the opioid epidemic, now is the time to mount a comprehensive prevention effort to help reduce opioid misuse among young people.

## Figures and Tables

**Table 1:** Sample Characteristics (*N* = 1434).

	*n*	Weighted % (95% CI)
Designated Market Areas		
Fort Wayne, IN	292	8.0 (7.0–9.1)
South Bend, IN	253	10.5 (9.2–12.0)
Greensboro, NC	214	18.4 (16.1–21.0)
Charlotte, NC	244	30.2 (27.1–33.5)
Knoxville, TN	237	13.0 (11.4–14.9)
Memphis, TN	194	19.8 (17.2–22.8)
Age category		
18–24	656	42.3 (39.3–45.4)
25–34	778	57.7 (54.6–60.7)
Gender		
Male	480	44.9 (41.7–48.1)
Female	924	53.8 (50.5–57.0)
Transgender or other gender identity	23	1.3 (0.8–2.1)
Race/ethnicity		
Non-Hispanic White	991	70.3 (67.3–73.2)
Non-Hispanic Black	178	19.7 (17.0–22.7)
Hispanic/Latinx	131	5.7 (4.6–7.0)
Non-Hispanic some other race	127	4.3 (3.5–5.2)
Perceived financial situation		
Cannot pay for basic expenses	67	4.6 (3.4–6.1)
Can pay for basic expenses with nothing left over	247	16.3 (14.1–18.7)
Can pay for basic expenses with a little left over	737	50.4 (47.2–53.6)
Can pay for basic expenses with more than a little left over	376	28.7 (25.9–31.8)
Sensation seeking (range = 1–5)		
Mean (SE)	2.91 (0.03)	(2.86–2.96)
Depression scale (range = 0–4)		
Mean (SE)	1.82 (0.03)	(1.77–1.88)
Anxiety and stress scale (range = 0–3)		
Mean (SE)	0.95 (0.02)	(0.90–1.00)
Number of COVID-19 related disruptions (range = 0–12)		
Mean (SE)	4.52 (0.07)	(4.38–4.65)
Misused opioids in past 30-days		
No	1368	95.3 (93.7–96.5)
Yes	63	4.7 (3.5–6.3)
Friend or family member misused opioids in past 6 months		
No	1128	78.2 (75.5–80.7)
Yes	306	21.8 (19.3–24.5)

**Table 2:** Campaign awareness overall and by age group.

	Overall (*n* = 1418)		18–24 Years (*n* = 648)		25–34 Years (*n* = 770)	
	*n* (Weighted %)	95% CI	*n* (Weighted %)	95% CI	*n* (Weighted %)	95% CI
Campaign awareness						
None	550 (44.1)	(40.9–47.3)	203 (36.4)	(32.0–40.9)	347 (49.7)	(45.3–54.1)
Low	437 (30.0)	(27.2–33.0)	203 (30.9)	(26.8–35.2)	234 (29.4)	(25.5–33.5)
High	431 (26.0)	(23.4–28.7)	242 (32.8)	(28.8–37.1)	189 (21.0)	(17.7–24.5)

**Table 3:** Results of logistic regression models examining association between campaign awareness and opioid knowledge, attitudes, and beliefs, controlling for DMA.

	Campaign-Targeted Outcomes					Outcomes Not Targeted by the Campaign	
	Opioid Dependence Can Happen in Just Five Days	Someone Like Me Could Become Dependent on Prescription Opioids	Risk Attitudes of Prescription Opioid Misuse	Look Up Information about the Opioid Epidemic	Talk to a Friend about the Opioid Epidemic	Using Medication to Treat Opioid Dependence Is Like Using It to Treat Chronic Diseases	Risk Attitudes of Illicit Opioid Misuse
Campaign awareness							
None	REF	REF	REF	REF	REF	REF	REF
Low	1.52 * (1.04, 2.24)	1.19 (0.81, 1.76)	1.01 (0.64, 1.58)	1.56 * (1.11, 2.21)	1.37 (0.96, 1.94)	1.05 (0.75, 1.47)	0.60 (0.26, 1.41)
High	2.47 *** (1.58, 3.87)	1.59 * (1.06, 2.40)	0.88 (0.54, 1.42)	1.75 ** (1.21, 2.54)	1.93 *** (1.33, 2.81)	0.96 (0.66, 1.40)	0.88 (0.35, 2.21)
Pre-campaign outcome †	5.42 *** (3.89, 7.54)	7.88 *** (5.63, 11.02)	4.99 *** (3.29, 7.57)	4.33 *** (3.27, 5.73)	4.58 *** (3.42, 6.13)	3.35 *** (2.51, 4.46)	4.16 * (1.18, 14.71)
Age category							
18–24	1.10 (0.78, 1.55)	0.78 (0.56, 1.09)	0.87 (0.60, 1.28)	0.92 (0.68, 1.24)	0.75 (0.56, 1.01)	0.87 (0.64, 1.17)	0.37 * (0.17, 0.80)
25–34	REF	REF	REF	REF	REF	REF	REF
Gender							
Male	REF	REF	REF	REF	REF	REF	REF
Female	1.29 (0.92, 1.80)	0.70 * (0.50, 0.98)	1.38 (0.95, 2.01)	1.27 (0.94, 1.71)	1.42 * (1.05, 1.93)	0.99 (0.73, 1.35)	1.52 (0.74, 3.10)
Transgender or other gender identity	1.43 (0.44, 4.65)	1.23 (0.43, 3.56)	2.24 (0.68, 7.41)	1.04 (0.31, 3.45)	1.34 (0.37, 4.83)	1.31 (0.50, 3.43)	2.77 (0.35, 21.87)
Sensation seeking	0.97 (0.78, 1.19)	1.08 (0.87, 1.35)	0.88 (0.69, 1.11)	1.10 (0.91, 1.33)	1.19 (1.00, 1.42)	1.11 (0.92, 1.34)	1.06 (0.69, 1.65)
Race/ethnicity							
Non-Hispanic White	REF	REF	REF	REF	REF	REF	REF
Non-Hispanic Black	0.66 (0.41, 1.07)	0.60 * (0.37, 0.97)	0.92 (0.52, 1.62)	1.52 (0.98, 2.37)	1.16 (0.75, 1.81)	0.57 * (0.35, 0.92)	0.92 (0.34, 2.51)
Hispanic/Latinx	0.71 (0.40, 1.27)	0.75 (0.43, 1.33)	1.04 (0.52, 2.10)	0.84 (0.49, 1.41)	1.29 (0.79, 2.11)	1.03 (0.61, 1.74)	0.77 (0.28, 2.15)
Non-Hispanic some other race	0.92 (0.56, 1.50)	0.78 (0.44, 1.36)	0.75 (0.38, 1.48)	1.44 (0.87, 2.40)	1.10 (0.63, 1.92)	0.77 (0.49, 1.21)	0.56 (0.18, 1.75)
Perceived financial situation							
Cannot pay for basic expenses	0.69 (0.34, 1.40)	0.87 (0.41, 1.85)	1.01 (0.40, 2.55)	1.01 (0.52, 1.98)	0.91 (0.44, 1.89)	0.64 (0.30, 1.37)	0.78 (0.16, 3.85)
Can pay for basic expenses with nothing left over	0.54 * (0.32, 0.91)	0.86 (0.53, 1.39)	1.36 (0.76, 2.43)	0.81 (0.53, 1.26)	0.79 (0.49, 1.25)	0.72 (0.46, 1.14)	1.05 (0.35, 3.20)
Can pay for basic expenses with a little left over	0.99 (0.66, 1.46)	1.29 (0.87, 1.92)	1.46 (0.95, 2.25)	1.07 (0.76, 1.50)	1.10 (0.77, 1.57)	1.07 (0.76, 1.52)	1.85 (0.80, 4.26)
Can pay for basic expenses with more than a little left over	REF	REF	REF	REF	REF	REF	REF
Depression scale	1.18 (0.91, 1.53)	1.25 (0.97, 1.62)	1.03 (0.77, 1.39)	0.96 (0.77, 1.21)	1.09 (0.86, 1.38)	1.10 (0.87, 1.38)	2.13 * (1.05, 4.31)
Anxiety and stress scale	0.98 (0.71, 1.36)	1.16 (0.86, 1.57)	0.69 * (0.50, 0.95)	1.17 (0.89, 1.53)	1.06 (0.80, 1.40)	1.29 (0.98, 1.70)	0.39 ** (0.19, 0.78)
Number of disruptions due to COVID-19	1.05 (0.96, 1.14)	1.15 *** (1.06, 1.26)	1.16 ** (1.06, 1.28)	1.08 * (1.01, 1.16)	1.11 ** (1.03, 1.19)	1.15 *** (1.07, 1.24)	1.27 * (1.04, 1.53)
Misused opioids in past 30-days	1.18 (0.50, 2.79)	1.05 (0.44, 2.48)	0.68 (0.30, 1.52)	0.84 (0.41, 1.70)	0.86 (0.41, 1.77)	0.92 (0.44, 1.92)	2.71 (0.45, 16.40)
Friend or family member misused opioids in past 6 months	1.37 (0.90, 2.10)	1.01 (0.67, 1.53)	1.43(0.90, 2.27)	1.72 ** (1.20, 2.46)	1.69 ** (1.16, 2.47)	0.85 (0.60, 1.21)	1.31 (0.58, 2.97)
County-level overdose death rates	1.00 (0.97, 1.02)	1.00 (0.98, 1.02)	1.01 (0.98, 1.03)	1.01 (0.99, 1.03)	1.00 (0.98, 1.02)	1.01 (0.99, 1.03)	0.98 (0.95, 1.02)
Designated market areas							
Fort Wayne, IN	1.15 (0.69, 1.91)	0.72 (0.45, 1.16)	1.13 (0.63, 2.03)	0.84 (0.54, 1.30)	0.63 * (0.41, 0.99)	0.78 (0.51, 1.21)	1.73 (0.56, 5.33)
South Bend, IN	0.90 (0.57, 1.41)	0.90 (0.56, 1.44)	1.07 (0.61, 1.88)	0.84 (0.55, 1.28)	0.84 (0.55, 1.29)	0.92 (0.60, 1.41)	2.21 (0.74, 6.60)
Greensboro, NC	0.92 (0.53, 1.58)	0.91 (0.54, 1.54)	0.66 (0.36, 1.23)	0.62 * (0.39, 0.98)	0.66 (0.41, 1.07)	1.05 (0.66, 1.69)	1.59 (0.55, 4.59)
Charlotte, NC	REF	REF	REF	REF	REF	REF	REF
Knoxville, TN	1.28 (0.63, 2.62)	1.18 (0.66, 2.11)	0.83 (0.40, 1.73)	0.76 (0.43, 1.34)	0.78 (0.44, 1.37)	0.63 (0.35, 1.11)	3.46 (0.88, 13.58)
Memphis, TN	1.20 (0.70, 2.07)	0.77 (0.45, 1.31)	1.00 (0.53, 1.87)	0.93 (0.58, 1.52)	0.91 (0.56, 1.49)	0.84 (0.52, 1.37)	1.48 (0.52, 4.20)

* *p* < 0.05; ** *p* < 0.01; *** *p* < 0.001; † Pre-campaign outcome refers to the dichotomized responses to the same item as the outcome measured with the post-intervention survey.

## Data Availability

The data presented in this study are available on request from the corresponding author.
